# Monomethyltransferase SET8 facilitates hepatocellular carcinoma growth by enhancing aerobic glycolysis

**DOI:** 10.1038/s41419-019-1541-1

**Published:** 2019-04-05

**Authors:** Xiangyuan Chen, Xiaowei Ding, Qichao Wu, Jie Qi, Minmin Zhu, Changhong Miao

**Affiliations:** Department of Anesthesiology, Fudan University Shanghai Cancer Center, Department of Oncology, Shanghai Medical College, Fudan University, shanghai, 200032 China

## Abstract

Hepatocellular carcinoma (HCC) is one of the most aggressive cancers worldwide. Despite such a public health importance, efficient therapeutic agents are still lacking for this malignancy. Most tumor cells use aerobic glycolysis to sustain anabolic growth, including HCC, and the preference of glycolysis often leads to a close association with poorer clinical outcomes. The histone methyltransferase SET8 plays crucial roles in controlling cell-cycle progression, transcription regulation, and tumorigenesis. However, it remains largely undefined whether SET8 affects the glucose metabolism in HCC. Here, we report that upregulation of SET8 is positively correlated with a poor survival rate in HCC patients. Both in vitro and in vivo studies revealed that SET8 deficiency conferred an impaired glucose metabolism phenotype and thus inhibited the progression of HCC tumors. By contrast, SET8 overexpression aggravated the glycolytic alterations and tumor progression. Mechanistically, SET8 directly binds to and inactivates KLF4, resulting in suppression of its downstream SIRT4. We also provided further evidence that mutations in SET8 failed to restrain the transactivation of SIRT4 by KLF4. Our data collectively uncover a novel mechanism of SET8 in mediating glycolytic metabolism in HCC cells and may provide a basis for targeting SET8 as a therapeutic strategy in HCC.

## Introduction

Liver cancer, one of the most common solid malignancies in the digestive system, is the second leading cause of cancer-related deaths worldwide^1^. Hepatocellular carcinoma (HCC) accounts for about 90% of all cases of primary liver cancer, with an estimating 800,000 new cases yearly. Overall, a 5-year survival rate is in desperate 18% for all patients, even worse for those with distant metastasis. The incidence and death rates of HCC continue to increase rapidly, despite advances made in cancer therapeutic approaches^2^. Environmental, genetic, and epigenetic factors are the main causes for HCC carcinogenesis, along with oncogene activation and tumor suppressor inactivation.

Cancer cells alter their metabolic phenotype to sustain the need for persisted proliferation, mainly by shifting from oxidative phosphorylation to aerobic glycolysis, also known as Warburg effect^3,4^. This progress is characterized by a marked increase in glucose uptake and flux through glycolysis and lactate production, even under normoxic conditions. The rewiring metabolic progress provides tumor cells with a diversion of glycolytic intermediates for the demand of biosynthesis of essential cellular components and maintenance of cellular redox homeostasis^5^. However, with such a great consumption in glucose, tumor cells put themselves in a condition of a harsh microenvironment and therefore must engage adoptive strategies to survive the metabolic stress.

SET8 (also known as KMT5A, Pr-Set7, and SETD8) is the only known monomethyltransferase of histone 4 at lysine 20 (H4K20me1) and has been reported to be involved in diverse biological processes, including DNA replication, DNA damage repair, cell-cycle progression, and transcription regulation^6,7^. During mitosis, the SET8 protein expression and H4K20me1 level are regulated in a ubiquitylation-mediated proteolysis manner, resulting in the highest expression during G2/M and early G1 phases, while almost absent in the S phase^8,9^. Besides H4K20, SET8 also displays robust enzymatic activity toward lysine residues of other nonhistone proteins, including p53 and PCNA^10–12^. However, the role of SET8 in transcription regulation can be ambiguous, functioning as both transcription activation and transcription repression^13–15^. Of note, a wealth of evidence has proposed that SET8 is overexpressed in many types of tumor issues and cell lines^7^. SET8 is implicated in cancer proliferation, migration, invasiveness, and oncogenesis, associated with a poor survival rate of cancer patients^16–18^. Recent evidence indicates that SET8-mediated H4K20 lysine methylation is involved in the metabolic gene expression^19^. A report also revealed that SET8 promoted glucose metabolism via stabilizing HIF1α in breast cancer^20^. Although the importance of SET8 in cell cycle and cell proliferation is well recognized, the role of SET8 in metabolism is beginning to be unraveled.

Here, we tend to investigate the role of SET8 in mediating glucose metabolism in hepatocellular carcinoma. Interestingly, we found that augmented expression of SET8 was frequently detected in hepatocellular carcinoma tissue and was significantly associated with a poor outcome. Furthermore, using gain- and loss of function, we found that SET8 facilitated aerobic glycolysis in HCC cancer cells, via enhanced expression of key enzymes and a glucose transporter in glycolysis. Both in vitro and in vivo experiments identified SET8 as a positive regulator of glucose metabolism. Mass spectrometry analysis revealed that SET8 interacted with KLF4^21^, which is reported to function as a tumor suppressor in various types of cancer, including HCC. In this study, we focused on the SET8/KLF4 signaling pathway in regulating aerobic glycolysis in HCC.

## Materials and methods

### Cell lines

Human hepatocellular carcinoma HCC-LM3 and MHCC97H cell lines were obtained from the Institute of Biochemistry and Cell Biology, Chinese Academy of Sciences, Shanghai, China. HEK293T cell line was purchased from American Type Culture Collection (Manassas, VA, USA). All the cell lines were cultured in the Dulbecco minimum essential medium (DMEM), supplemented with 10% fetal bovine serum (Gibco Invitrogen, Grand Island, NY, USA) containing 1% penicillin–streptomycin at 37 °C in a humidified atmosphere with 5% CO_2_.

### Mass spectrometry

Whole-cell protein lysates were extracted 48 h after transfection with flag-tagged SET8. Endogenous IP was performed as described above. Beads were washed with flag peptide. Supernatants with loading buffer were subjected to SDS–PAGE electrophoresis. Silver staining was determined with Fast Silver Stain Kit (Beyotime Biotechnology, Shanghai) according to the manufacturer’s instructions. Stained gel bands were retrieved and analyzed by mass spectrometry using a high-performance liquid chromatography system (1260 series, Agilent Technologies) and a mass spectrometer (Agilent 6460, Agilent Technologies).

### Chromatin immunoprecipitation assay

Chromatin immunoprecipitation (ChIP) assays were conducted with Simple ChIP Plus Sonication Chromatin IP Kit (Cell Signaling Technology, MA) according to the manufacturer’s instructions. Briefly, cells (1 × 10^7^) were fixed with 1% formaldehyde for 10 min at room temperature to cross-link DNA and proteins. Glycine was then added to stop the cross-linking. Chromatin was sheared using Microson Ultrasonic Cell Disruptor XL (Misonix) with 16 cycles of sonication (15 s each, 2-min rest; amplitude = 10, power = 15 W). Ten-microliter sonication solution was taken out from each sample as the input control, and the remaining was incubated with anti-KLF4 (Abcam, USA) or anti-H4K20me1 (Abcam, USA), or histone H3-positive control, or IgG negative control at 4 °C overnight. Immunoprecipitates were bound to protein G magnetic beads, and the DNA–protein cross-link was reversed at 65 °C for 2 h. DNA was purified and enrichment of DNA sequences was detected using qPCR. SIRT4 oligonucleotide sequences for PCR primers were forward 5′-GAAGAGATGGGATCTCACTTTGTC-3′ and reverse 5′-GTAGACAACCAGAACTGCCGCTCT-3′.

### Animal model of tumor growth

Animal experiments were performed in compliance with recommendations of the Guide for the Care and Use of Laboratory Animals of Fudan University. Five-week-old female BALB/c nude mice were purchased from Shanghai SLAC Laboratories and nurtured in a temperature-controlled environment under a 12-h light/dark time cycle. Mice (five per group) were injected with 10^6^ HCC-LM3 or SET8-overexpression cells or shSET8 cells in the right flanks. Six weeks after tumor implantation, mice were subjected to MicroPET/CT scanning. Tumor volume was measured every 3 days and calculated using the following formula: (length × width^2^)/2. Harvested tumors were weighed and then used for IHC analysis.

### Statistical analysis

Data are presented as mean ± SD/SEM and analyzed using unpaired Student’s *t* test and ANOVA (SPSS Software). Experiments were performed in triplicate. A value of *p* < 0.05 was considered statistically significant, and all tests were two sided.

## Results

### SET8 is overexpressed and associated with poor prognosis of hepatocellular carcinoma

**Fig. 1:**
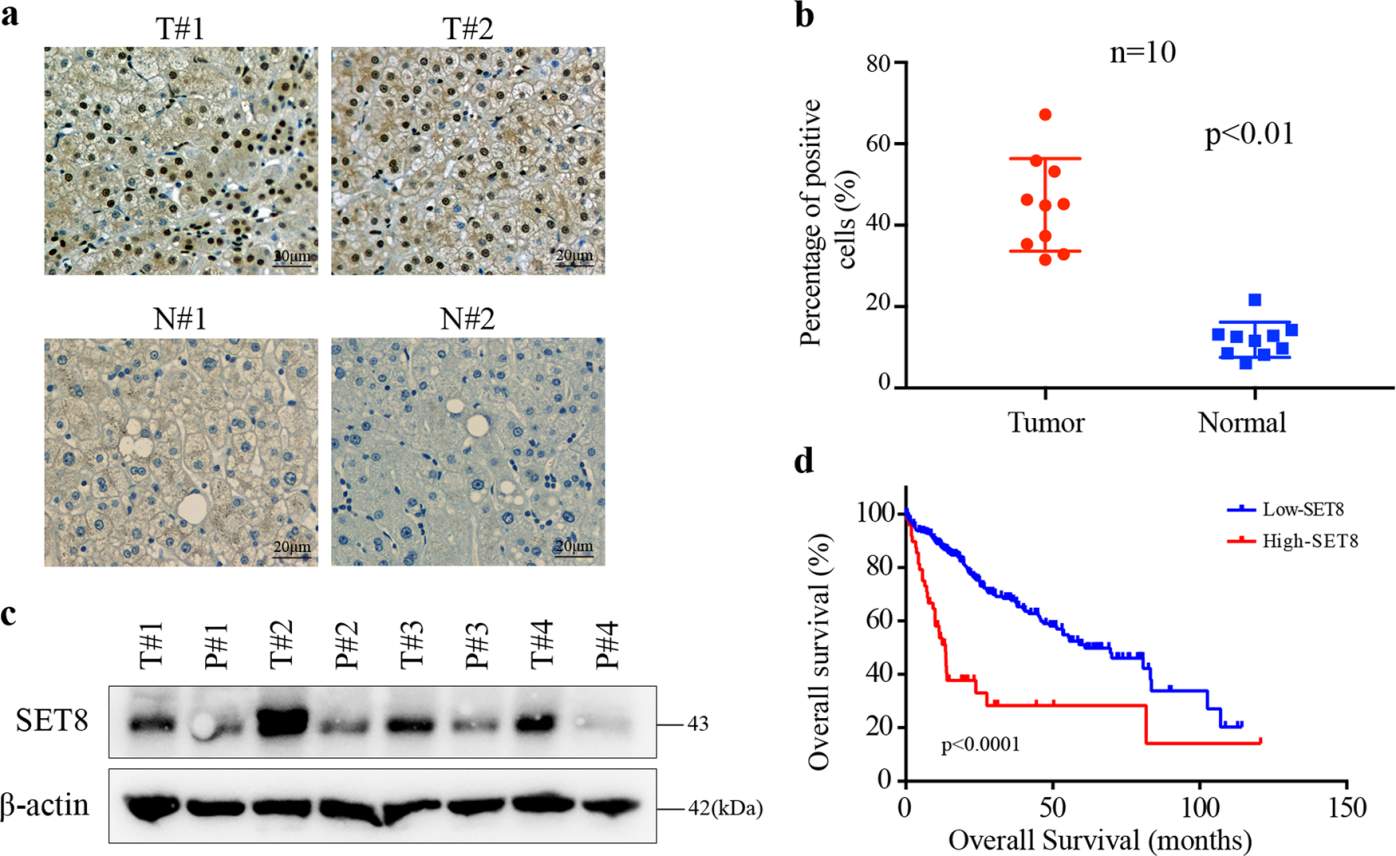
SET8 expression levels predicted survival in HCC patients. **a** Representative images of SET8 expression in HCC specimens and its adjacent normal liver tissue specimens. Nuclei (blue) were stained with hematoxylin. Scale bar, 20 μm. **b** Analysis of SET8 expression in HCC specimens and its adjacent normal liver tissue specimens (*n* = 10, *p* < 0.01). **c** Protein expression of SET8 in paired HCC specimens and its adjacent normal liver tissue specimens by western blot analysis. **d** Kaplan–Meier OS survival curve based on SET8 expression in the TCGA dataset of HCC. Data are shown as mean ± SD of three independent experiments. ^*^P < 0.05

To determine the roles of SET8 in hepatocellular carcinoma pathogenesis, we first analyzed SET8 expression in the 10 paired primary carcinomas of liver and tumor-adjacent tissue specimens. We observed SET8-positive staining in tumor cells, whereas no or very low staining was detected in the nonmalignant specimens (Fig. 1a, b). Furthermore, western blot analysis confirmed the expression of SET8 in paired normal liver tissue and HCC specimens (Fig. 1c). To validate the effects of SET8 expression on the patients’ survival with HCC, we assessed survival rates in the TCGA dataset of HCC. On the basis of SET8 expression, patients were divided into two groups using the minimum *P*-value approach^22^. Notably, a higher SET8 expression was significantly associated with a poorer overall survival rate in the TCGA dataset (Fig. 1d). Collectively, these data suggested that the expression of SET8 was enhanced in HCC tumor specimens and a higher SET8 expression was significantly associated with poor survival in patients with HCC.

### SET8 facilitates cell proliferation and regulates apoptosis to assist HCC cell survival

**Fig. 2:**
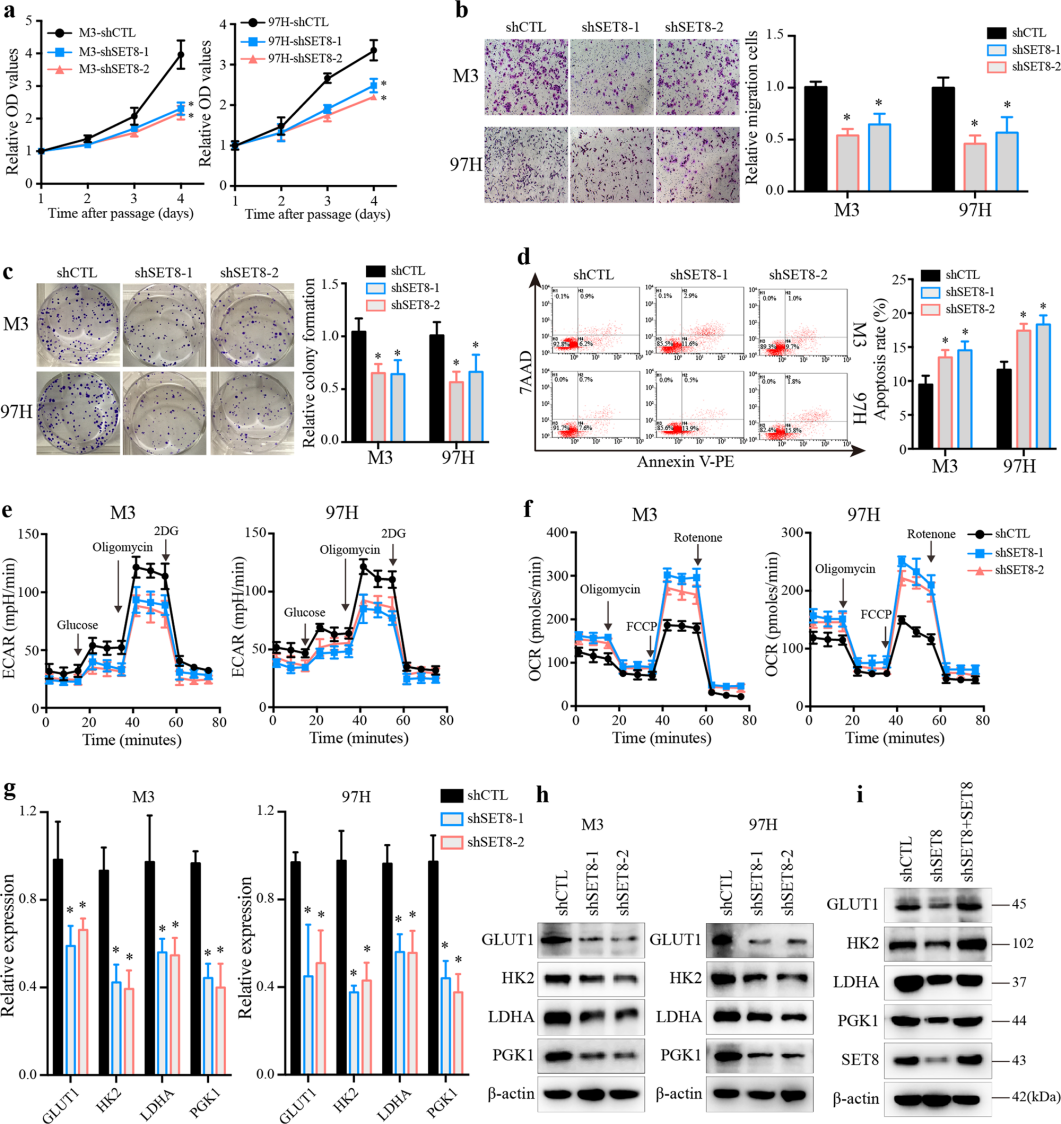
Inhibition of SET8 impaired cell proliferation and reduced glycolysis in HCC cells. **a** Assessment of cell growth via CCK-8 assay in control (shCTL) and SET8 knockdown (shSET8) M3 (left) and 97H (right) cells. **b** Migration assay of shCTL and shSET8 cells using a transwell system. Upper, M3 cells; down, 97H cells. **c** Colony-formation assay performed in shCTL and shSET8 cells, and numbers of colonies were counted 14 days later. **d** Flow-cytometry analysis using Annexin V and 7AAD staining to determine the apoptosis rate. Upper, M3 cells; down, 97H cells. **e**, **f** Analysis of the extracellular acidification rate (ECAR) and oxygen consumption rate (OCR) in M3 (left) and 97H (right) cells with either control shRNA or SET8 shRNA. **g**, **h** The expression levels of genes involved in glucose metabolism in the indicated HCC cell lines with either SET8 knockdown or control shRNA detected by real-time PCR and western blot analysis. **i** SET8 shRNA-infected cells were transfected with the SET8 plasmid for 48 h, and cells were then subjected to western blot analysis. Data are shown as mean ± SD of three independent experiments. ^*^P < 0.05

To investigate the biological significance of SET8 in HCC cells, both loss-of- and gain-of-function approaches were employed. We established HCC cell lines (HCC-LM3, M3 and MHCC97H, 97H) stably expressing SET8 or SET8 knockdown with their own vector control, respectively. Overexpression of SET8 was confirmed by RT-PCR and western blot analysis (Supplementary Fig. 1a). The SET8 mRNA and protein were reduced by two small hairpin RNAs with a similar knockdown efficacy (Supplementary Fig. 1b). Consistent with previous reports in cancer cell lines^16,17^, knockdown of SET8 greatly reduced cell proliferation, clonogenicity, and migration (Fig. 2a–c), while ectopic expression of SET8 produced the opposite effects (Supplementary Fig. 1c–e). Tumor cells evolve multiple strategies to avoid apoptosis to sustain the malignant state^3^. Therefore, we tended to explore the role of SET8 on apoptosis of HCC cells. As shown in Fig. 2d, induced apoptosis was observed upon SET8 silencing. Consistently, ectopic expression of SET8 reduced apoptosis in HCC cells (Supplementary Fig. 1f). These data demonstrated that SET8 plays crucial roles in HCC cell proliferation and migration.

### Reduced glycolysis and enhanced mitochondrial respiration in SET8-deficient cells

Altered metabolic activities have been proposed as a hallmark of cancer^3,23^. Tumor cells favor aerobic glycolysis as a source of energy and macromolecular precursors to support the acquisition and maintenance of the malignant state. Therefore, to evaluate the potential function of SET8 on glucose metabolism, we examined whether SET8 deficiency would affect the extracellular acidification rate (ECAR) and oxygen consumption rate (OCR) using Seahorse-XF Extracellular-Flux Analyzer. Importantly, SET8 deficiency markedly decreased the glycolytic capacity (Fig. 2e), but increased the mitochondrial function of oxidative phosphorylation (Fig. 2f) in HCC cell lines. Correspondingly, we observed a significant decrease in the mRNA and protein level of key regulators (GLUT1, HK2, LDHA, and PGK1) involved in glucose metabolism upon SET8 silencing (Fig. 2g, h). In line with the above observation, overexpression of SET8 led to the opposite effects (Supplementary Fig. 2a–d). To further support the role of SET8 in HCC cell glucose metabolism, we overexpressed SET8 in SET8-deficient (sh1) cells (Fig. 2i). Notably, enhanced SET8 expression in SET8-deficient cells reversed the downregulation of glucose metabolism genes (Fig. 2i). To further confirm the results, we constructed a dominant negative mutant SET8^R295G^ palsmid^24^. Further analysis revealed no obvious effect upon re-expression of the mutant SET8 (SET8^R295G^) in SET8 knockdown cells (Supplementary Fig. 2e). Together, our data demonstrated that SET8 resulted in a cellular metabolic shift from oxidative phosphorylation to glycolysis.

### SET8 is required for tumor growth

**Fig. 3:**
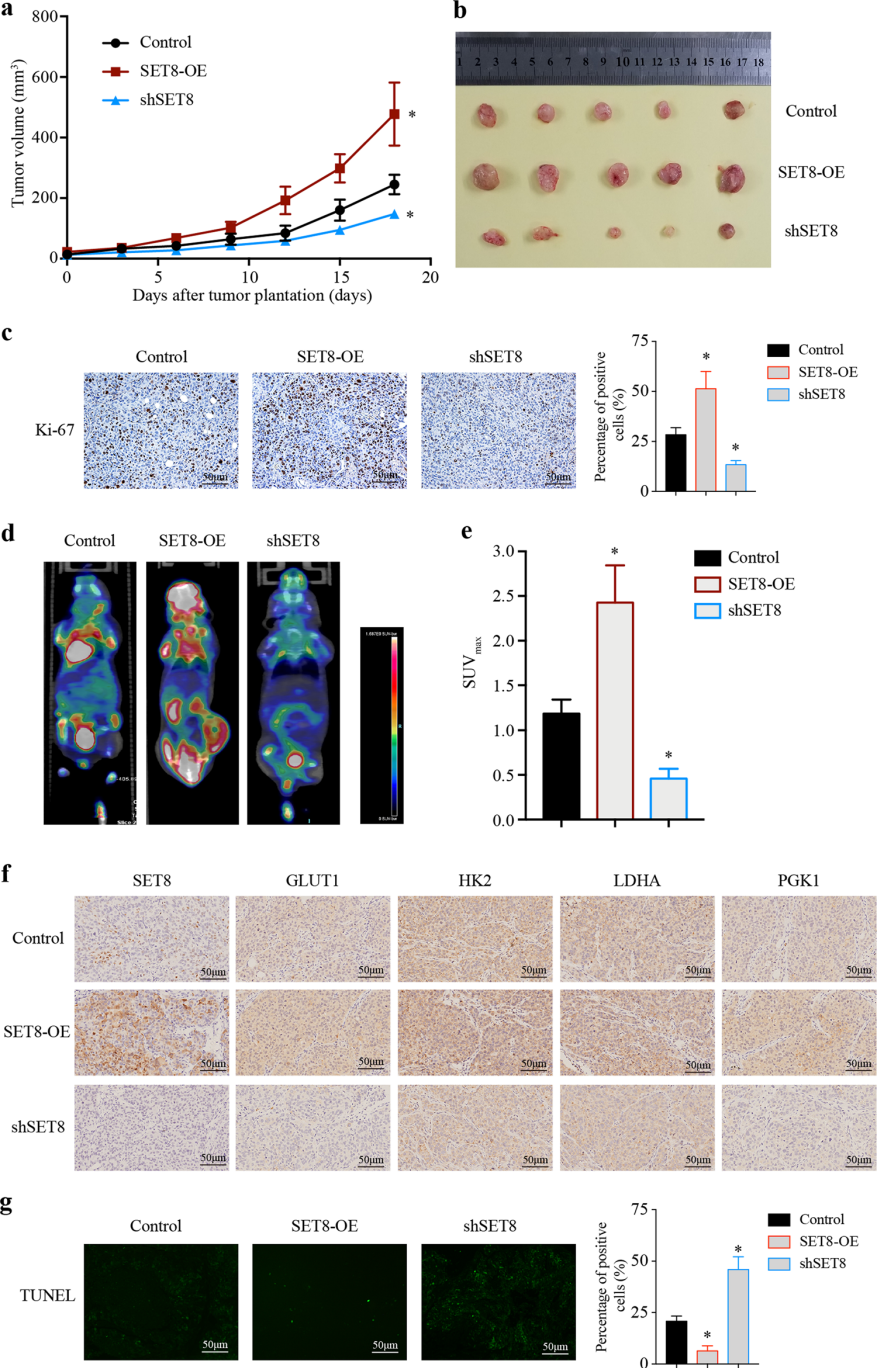
SET8 promoted glucose uptake and tumorigenicity in vivo. **a**, **b** Tumor growth following subcutaneous injection in the right flanks of Balb/c nude mice with 97H control cells, 97H-shSET8 cells, or 97H-SET8 cells (*n* = 5). **c** Immunostaining of Ki-67 in xenograft tissues. **d** Mice were imaged 60 min following intravenous (i.v.) injection of ^18^F-FDG by microPET/CT. Mice were fasted one night before detection. **e** Ratio of SUV_max_ of labeled ^18^F-FDG incorporation in the control group, shSET8 group, and SET8 group. **f** Immunostaining of SET8 and key glycolytic genes in tumors from the control group, shSET8 group, and SET8 group. **g** Cell apoptosis in xenograft tissues by TUNEL assay. Representative images of the immunostainings at ×20 magnification are shown. Scale bar, 50 μm

The above data prompted us to further examine the function of SET8 in vivo. Therefore, we subcutaneously injected 97H cells into the Balb/c nude mice to assess the ability of these cells to form tumors in vivo. In line with the in vitro results, downregulation of SET8 inhibited tumor growth, with fewer Ki-67 positive cells, while SET8 overexpression promoted tumor growth, with more Ki-67 positive cells (Fig. 3a–c). To determine the impact of SET8 on the glucose metabolic phenotype in vivo, we assessed the ^18^F-fluorodeoxyglucose (FDG) uptake in tumors using a microPET/CT system to monitor glucose metabolism. We observed that tumors formed by SET8-silencing cells displayed a pronounced reduction in glucose uptake (Fig. 3d, e). Conversely, SET8- overexpressing cells formed tumors and exhibited an increased glucose uptake (Fig. 3d, e). Analysis of key glycolytic genes in tumors by immunohistochemistry revealed similar results with in vitro experiments (Fig. 3f). Furthermore, the TUNEL assay showed that downregulation of SET8 resulted in increased cell apoptosis, while SET8 overexpression led to the opposite effect (Fig. 3g). These data suggested that SET8 promotes glucose uptake and tumorigenicity of HCC cells in the mouse model.

### SET8 mediates metabolic reprogramming by suppressing SIRT4

**Fig. 4:**
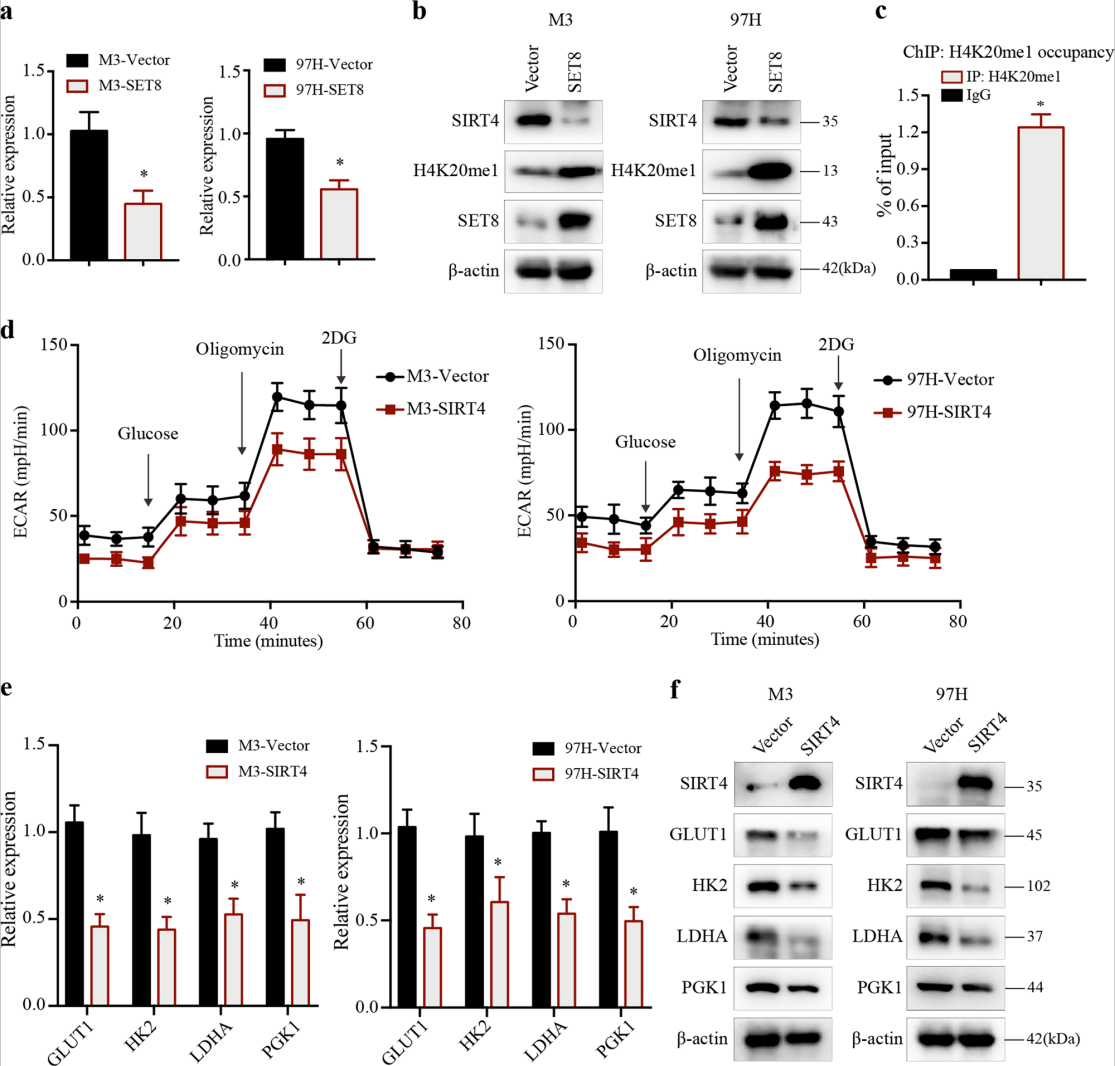
SET8 promoted aerobic glycolysis by suppressing SIRT4. **a** Relative mRNA level of SIRT4 in the indicated HCC cell lines with either SET8 overexpression or control vector detected by real-time PCR. **b** Protein levels of SIRT4, H4K20me1, and SET8 in the indicated HCC cell lines with either SET8 overexpression or control vector detected by western blotting. **c** ChIP assay of H4K20me1 occupancy at the promoter region of SIRT4. Normal IgG was used a control, and 1% of the total cell lysates was subjected to PCR before immunoprecipitation (input control). **d** Analysis of the extracellular acidification rate (ECAR) in the indicated HCC cell lines with either SIRT4 overexpression or control vector. **e**, **f** The expression levels of genes involved in glucose metabolism in the indicated HCC cell lines with either SIRT4 overexpression or control vector detected by real-time PCR and western blot analysis. Data are shown as mean ± SD of three independent experiments. ^*^P < 0.05

SIRT4 was initially characterized by its enzymatic activity on ADP-ribosylate glutamate dehydrogenase (GLUD1) that converts glutamate (derived from glutamine) into the intermediate α-ketoglutarate^25^. SIRT4 has been recently reported to inhibit the pyruvate dehydrogenase complex (PDH), and thereby implicated a role in aerobic glycolysis^26^. To this end, we reasoned that SET8 might mediate the expression of SIRT4 in regulating glucose metabolism. Strikingly, SIRT4 mRNA and protein levels diminished in HCC cells stably expressing SET8 (Fig. 4a, b). Conversely, H4K20me1 level was significantly increased in SET8-overexpressing cells (Fig. 4b). Next, to identify whether SIRT4 is targeted by SET8, we examined the genome-wide H4K20me1 distribution in HCC cells. Chromatin immunoprecipitation (ChIP) assay revealed that H4K20me1 was enriched at the SIRT4 promoter region (Fig. 4c). These results suggested that SET8 functions as a repressor for SIRT4.

We next determined the importance of SIRT4 for the metabolic phenotype in HCC cells. HCC cells were transfected with SIRT4 or control vector (Fig. 4f). ECAR analysis revealed that SIRT4-overexpressing cells exhibited a decreased glycolytic capacity compared with control cells (Fig. 4d). The mRNA and protein expression of key enzymes in glycolysis were also decreased when SIRT4 was overexpressed (Fig. 4e, f), consistent with the results of reduced glycolysis in SIRT4-overexpressing cells. Conversely, SIRT4-silencing cells displayed an opposite metabolic phenotype (Supplementary Fig. 3a–c). To validate that SIRT4 plays a critical role in this phenotype, we decided to elevate SIRT4 in SET8-overexpressing cells and to test whether enhanced SIRT4 could rescue the metabolic abnormalities observed in these cells. Notably, enhanced SIRT4 was sufficient to revert the increased expression of key glycolytic genes in SET8-overexpressing cells (Supplementary Fig. 3d). To further address the point, we silenced SIRT4 in SET8 knocking-down cells, and found that SIRT4 silencing recovered ECAR and cell proliferation, but counteracted increased apoptosis in SET8 knocking-down cells (Supplementary Fig. 3e–g). Taken together, our data suggested that enhanced aerobic glycolysis observed in SET8-overexpressing cells is a consequence of SIRT4 inactivation.

### SET8 interacts with KLF4

**Fig. 5:**
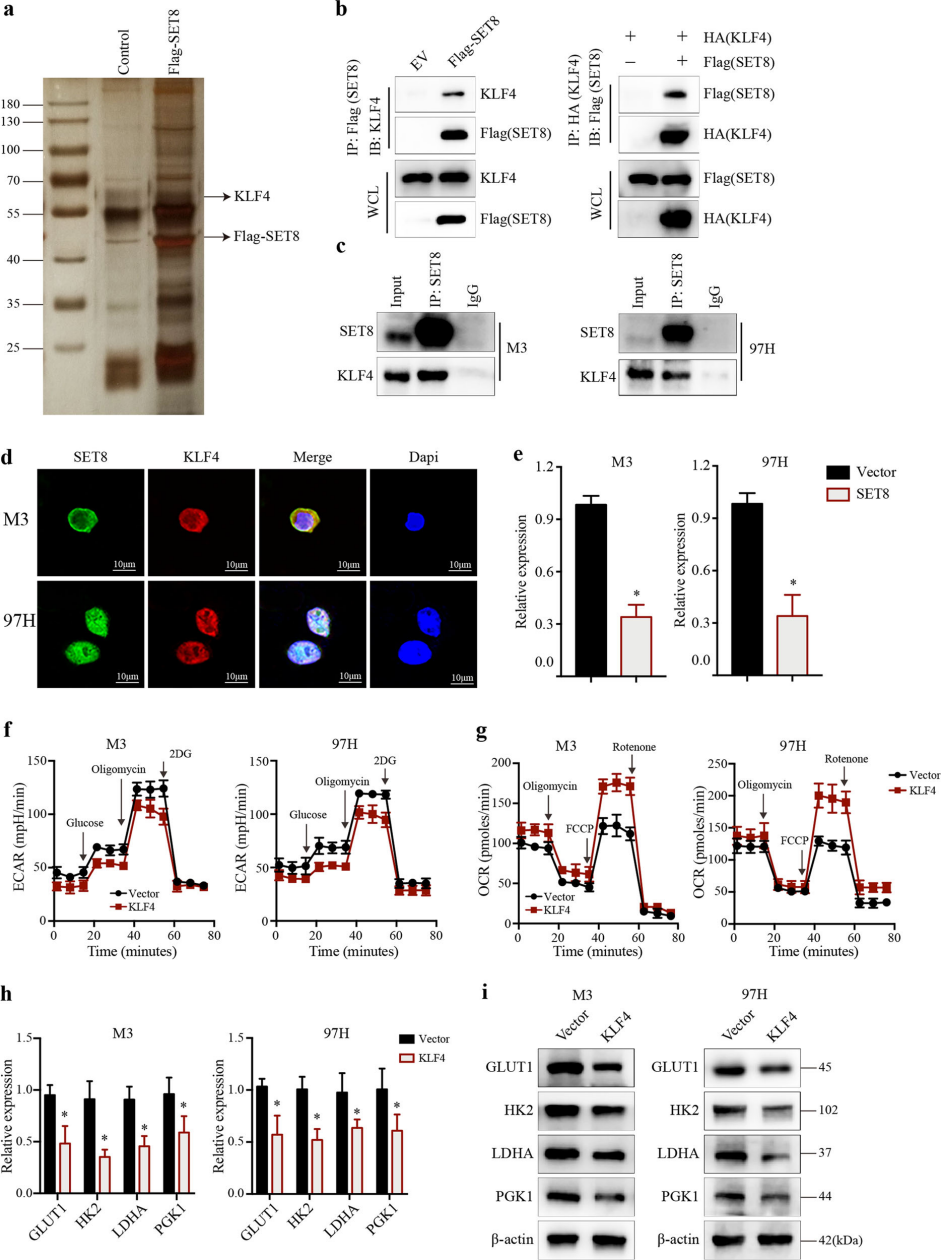
Identification of interaction between SET8 and KLF4. **a** 97H cells were infected with adenovirus-expressing GFP (Ad-GFP) or SET8 tagged with Flag epitope (Ad-SET8). Cells were harvested, and nuclear extracts were sequentially immunoprecipitated with Anti-FLAG (M2 agarose). The SET8-associated proteins were detected by SDS/PAGE and silver staining. **b** HEK293T cells were ectopically expressed with the indicated plasmids (Flag-SET8 and HA-KLF4). Extracts were immunoprecipitated with the antibody against Flag tag (IP: Flag-SET8/IB: KLF4) or KLF4 (IP: HA-KLF4/IB: Flag-SET8). **c** Validation of SET8–KLF4 interaction in vivo. Endogenous interaction between SET8 and KLF4 was observed in HCC cells measured by immunoprecipitation. **d** Colocalization of SET8 and KLF4 in HCC cells detected by confocal microscopy. **e** mRNA level of KLF4 detected in SET8-overexpressing cells. **f**, **g** Analysis of extracellular acidification rate (ECAR) and oxygen consumption rate (OCR) in M3 (left) and 97H (right) cells with either KLF4 overexpression or control vector. **h**, **i** The expression levels of genes involved in glucose metabolism in the indicated HCC cell lines with either KLF4 overexpression or control vector detected by real-time PCR and western blot analysis. Data are shown as mean ± SD of three independent experiments. ^*^P < 0.05

To gain insights into the mechanism by which SET8 promotes aerobic glycolysis, we took a biochemical approach to identify further SET8-interacting proteins. The purified SET8 complex was then resolved by SDS–PAGE followed by silver staining. The differential protein bands between the two groups were isolated and subjected to mass spectrometry^20,27^. Our efforts led to the identification of several unidentified interactors, including the transcription factor Krüppel-like factor 4 (KLF4) (Fig. 5a). Co-immunoprecipitation experiments confirmed that SET8 physically interacted with KLF4 (Fig. 5b). Furthermore, we also tested that SET8 coprecipitated with KLF4 in HCC cell lines (Fig. 5c). Finally, we demonstrated that SET8 and KLF4 are colocalized using confocal microscopy (Fig. 5d). We next tested whether SET8 affects KLF4 expression. We observed that SET8 overexpression resulted in a significant drop of KLF4 in both mRNA and protein levels (Fig. 5e; Supplementary Fig. 4a).

### KLF4 represses altered glucose metabolism and tumor progression in HCC cells

The transcription factor Krüppel-like factor 4 (KLF4) plays an ambivalent role in tumorigenesis^28^. Recently, KLF4 has been characterized as a potent tumor suppressor in HCC^29^. However, little is known about the role of KLF4 in HCC glucose metabolism. Therefore, we sought to assess whether KLF4 affects glucose metabolism in HCC. We established HCC cell lines stably expressing KLF4 or control vector (Supplementary Fig. 4b). Consistent with a previous report^30^, an impaired glycolysis in KLF4-overexpressing cells was observed (Fig. 5f). OCR analysis revealed that KLF4-overexpressing cells exhibited an increased mitochondrial oxygen consumption rate (Fig. 5g). In addition, we compared the expression of key glycolytic genes in KLF4-overexpressing cells with control ones. Accordingly, the mRNA and protein expression of glycolytic genes were significantly inhibited when KLF4 was overexpressed (Fig. 5h, i). Using two independent shRNAs against KLF4, we observed that depletion of KLF4 leads to an increased ECAR level and decreased OCR level in HCC cells (Supplementary Fig. 4c, d). Moreover, the level of key glycolytic genes was also increased in KLF4-deficient cells (Supplementary Fig. 4e, f). As SET8 interacted with KLF4, we tended to see if KLF4 affected the effect of SET8 in hepatocellular carcinoma cells. By downregulating KLF4 in SET8-silencing cells, we observed that downregulation of KLF4 was able to block the decrease in ECAR and cell proliferation, while abrogating the increase in OCR and cell apoptosis (Supplementary Fig. 4g–j). Taken together, our results revealed that KLF4 plays a role in redirecting carbohydrate flux from glycolysis to mitochondrial respiration.

Inactivation of KLF4 has been reported to be associated with the growth and survival of various cancer cells^31,32^. Next, we studied whether KLF4 functions in the biology of HCC malignancy. Indeed, wide-type KLF4 greatly reduced cell proliferation, colony formation, and cell migration (Supplementary Fig. 5a–c), but increased cell apoptosis (Supplementary Fig. 5d). Conversely, KLF4 deficiency conferred increased cell growth, colony formation, and cell migration, and alleviated cell apoptosis (Supplementary Fig. 5e–h).

### SET8 mediates transcriptional activation of SIRT4 by KLF4 in HCC cells

**Fig. 6:**
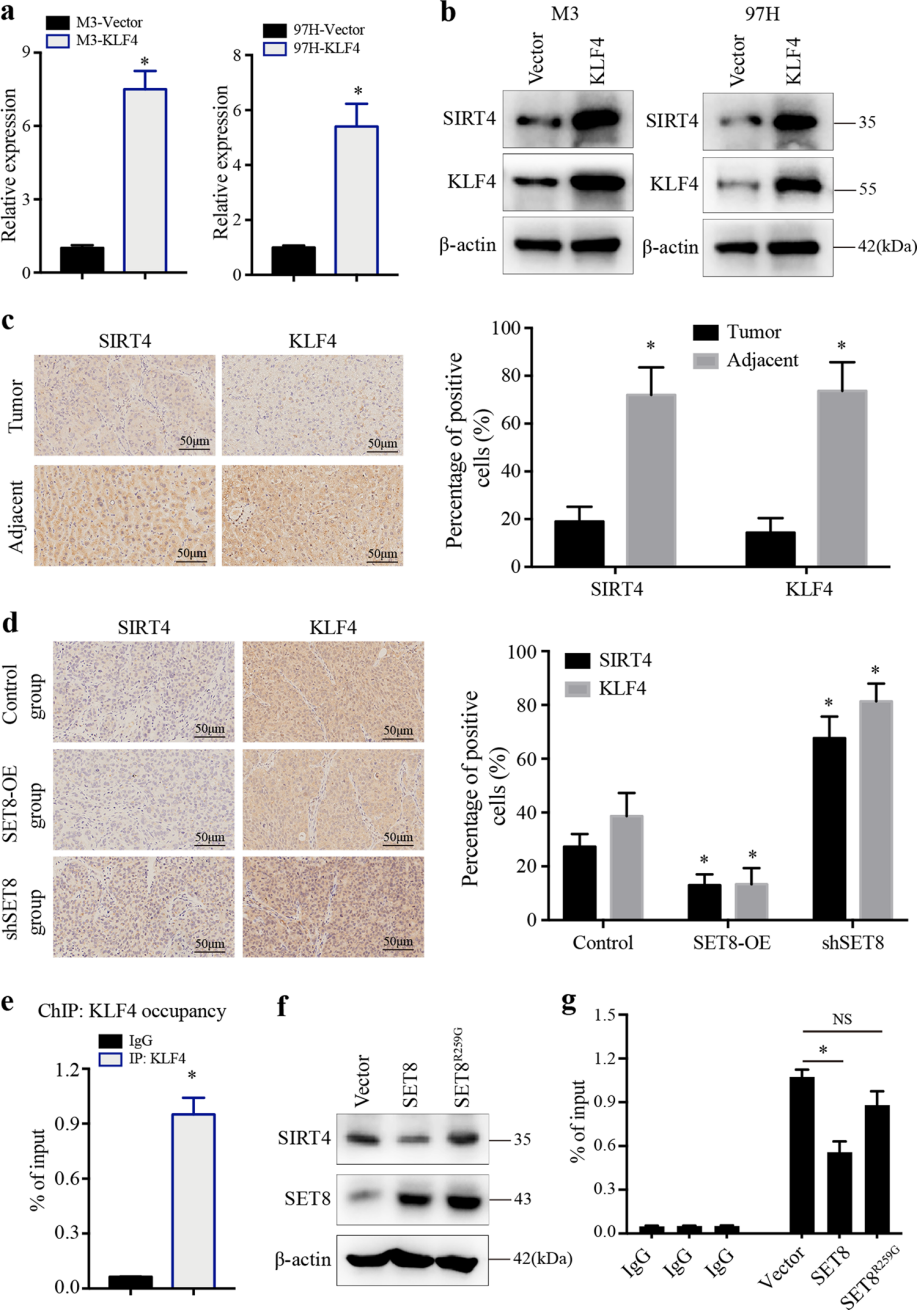
SET8 regulated SIRT4 expression in a KLF4-dependent manner. **a**, **b** The RNA and protein levels of SIRT4 detected by real-time PCR and western blotting in KLF4-overexpressing cells. **c**, **d** Representative IHC images of SIRT4 and KLF4 staining in paired HCC tumor tissues and mice model tumor tissues. Representative images of the immunostainings at ×20 magnification are shown. Scale bar, 50 μm. **e** KLF4 occupancy of the SIRT4 promoter detected by the ChIP assay. **f** Cells were transfected with the control vector, SET8 or the dominant negative mutant form SET8 mutant plasmid (SET8^R25G^). Extracts were analyzed for western blot analysis. **g** SET8 decreased the KLF4 occupancy of the SIRT4 promoter, whereas SET8^R25G^ had little influence. Data are shown as mean ± SD of three independent experiments. ^*^P < 0.05

Our results thus far indicate that KLF4 might function as a repressor in HCC cell glycolysis, and in the absence of KLF4, glycolysis is enhanced and the mitochondrial oxygen consumption is inhibited. KLF4 has been reported to be a transcription activator or repressor for its responsive genes^33^. Therefore, we further investigated the effects of an altered KLF4 expression on SIRT4 expression in HCC cells. As shown in Fig. 6a, b, overexpression of KLF4 led to an increased expression of both mRNA and protein levels of SIRT4. To further examine the regulatory effect of KLF4 on SIRT4 expression, we detected the expression of KLF4 and SIRT4 in human HCC tissue specimens and the mouse model tissue specimens. Statistical analysis revealed that the expression of SIRT4 was positively correlated with the expression of KLF4 in the two cohorts, and both SIRT4 and KLF4 expression were decreased in tumor tissues (Fig. 6c, d). In order to gain further insight into the regulation of SIRT4 by KLF4, ChIP assay was performed using an antibody to KLF4. Indeed, we found that KLF4 bound directly to the promoter of SIRT4 (Fig. 6e). To validate whether SET8 regulated SIRT4 expression in a KLF4-dependent manner, we constructed a dominant negative mutant form of SET8 (SET8^R259G^). Interestingly, cells transfected with SET8^R259G^ obtained no obvious change in the SIRT4 level, compared with control cells (Fig. 6f). Consistently, the ChIP assay revealed that the introduction of SET8 maintained high levels of H4K20me1 at the promoter region of SIRT4, thereby inhibiting transcription, despite the presence of KLF4, whereas SET8^R259G^ had little effect (Fig. 6g). Collectively, these results suggested demonstrated that SET8 promoted glucose reprogramming in HCC cells via regulating the KLF4/SIRT4 axis.

## Discussion

Altered metabolism, recognized in the mid-1990s by Otto Warburg^34^, is now accepted as one of the fundamental hallmarks of cancer^3^. Tumor cells rely on an inefficient glycolytic pathway for generation of ATP, even in the presence of oxygen, necessitating increased glucose consumption to maintain proliferation and growth, termed as “Warburg effect”. Augmented glycolysis and the concomitant increase in glucose consumption by malignant tumors is used clinically to diagnose and monitor treatment responses of cancers by imaging the uptake of fluorine-18 fluorodeoxyglucose (^18^F-FDG) with positron emission tomography (PET)^35^. Emerging evidences indicate that many cancers, including HCC, display an aerobic glycolytic phenotype, resulting in tumor progression and thereby affecting the clinical outcomes and clinicopathologic characteristics of cancer patients^36,37^. Despite the widespread appreciation and clinical application, how the Warburg effect is regulated in cancer remains largely unclear.

In this study, we demonstrate that SET8 might be a driving factor in HCC development by reprogramming glucose metabolism from mitochondrial oxygen phosphorylation to aerobic glycolysis. We first provided evidence that SET8 is highly expressed in HCC tumor tissues and from the TCGA cohort, we demonstrate that SET8 is positively correlated with overall survival and is an independent predictor in HCC patients. Mechanistically, SET8 directly or indirectly binds to and inactivates KLF4, thus inhibiting its downstream factor SIRT4. Serving as a co-regulator of SIRT4, SET8 sustains the survival of HCC cells both in vitro and in vivo by promoting glucose metabolism and thereby could be a potential therapeutic target in treatment of the HCC patients.

SET8, the only known methyltransferase for H4K20 monomethylation, has been implicated in a diverse array of biological processes, such as transcriptional regulation^6,7,14^. Notably, elevated expression levels of SET8 were found in multiple tumor cells and tissues, including HCC, closely correlated with a poor survival in cancer patients^38,39^. Consistently, we observed an increased SET8 expression in human HCC tissues compared with its paired adjacent tissues, and is associated with a reduced overall survival rate in HCC patients from the TCGA cohort. Moreover, the expression of SET8 is positively correlated with expression of glycolytic genes in HCC cells, supporting its relevance for the glycolytic phenotype of HCC cells. In line with our observation, SET8 is reported to coordinate with HIF1α to modulate glucose metabolism in breast cancer cells^20^. A recent study also reveals a role of SET8 in lipid metabolism in papillary thyroid carcinoma^40^, suggesting that SET8 might be critical in cancer metabolic reprogramming. Establishing the role of SET8 as a pro-survival factor regulating the Warburg effect, we demonstrate that SET8 deficiency impaired the aerobic glycolytic phenotype and survival of hepatoma cells both in vitro and in vivo. Thus, we reasoned that SET8 may promote HCC initiation and maintenance at least partly through upregulation of glucose metabolism.

SIRT4, a mitochondrial sirtuin, is revealed to play roles in many important processes, particularly in glutamine and fatty acid metabolism^41^. Many studies have established a tumor-suppressive role of SIRT4 in cancer^42^. Accordingly, we observed that SIRT4 staining was reduced in HCC tissues and its expression resulted in decreased cell proliferation and migration. Given that SIRT4 exhibited a role in inhibiting pyruvate dehydrogenase (PDH) activity^26^, it is tempting to speculate that SIRT4 might also be involved in the glycolytic process. To this end, we examined the effect of SIRT4 on glycolysis in HCC cells. Our data demonstrated that enhanced SIRT4 expression led to an impaired aerobic glycolysis by decreasing the expression of key glycolytic genes, further supporting the function of SIRT4 in maintaining cellular metabolic hemostasis.

The observation of the connection between SIRT4 and the Warburg effect led us to further the study by exploring whether SET8 promoted the Warburg effect by regulating SIRT4 expression. Previous studies have provided evidences that support the role of SET8 and H4K20me1 in gene regulation^13,14^. Here we observed that a global amount of H4K20me1 was increased, whereas the expression level of SIRT4 was decreased under the upregulation of SET8. Previous studies suggested that SET8 functions via inducing increased expression of H4K20me1 marks and subsequent recruitment to its targeted genes^13,14,19,43,44^. For that, SET8 mainly regulates gene transcription via H4K20me1; we therefore detected the distribution of H4K20me1 at the promoter of SIRT4. Using a ChIP assay, we observed an occupancy of H4K20me1 at the promoter region of SIRT4. Thus, it is likely that SET8 exerted a transcriptional repression effect on SIRT4 via H4K20me1. Consequently, we found that the abnormally activated glycolytic phenotype caused by SET8 overexpression was partially reversed by co-expression of ectopic SIRT4, suggesting SIRT4 as a mediator of metabolic effects triggered by SET8 overexpression. However, how H4K20me1 is implicated in the transcriptional repression requires further investigation.

KLF4, a member of the zinc finger transcription factor family, is reported to participate in various biological processes, including cell differentiation and proliferation^45^. In this study, we identified KLF4 as an interactor with SET8 using mass spectrometry analysis. Co-IP and immunofluorescence analysis further validated the interaction and colocalization between KLF4 and SET8. Expression of KLF4 has been associated with both tumor promotion and suppression. Numerous studies have depicted a potent protective role of KLF4 in various tumors^46,47^. Conversely, other reports found that KLF4 expression is increased in breast cancer, oral cancer, and skin squamous cancer, suggesting that KLF4 is crucial for the progression of these tumors^48–50^. Similar to previous findings^51^, our results supported that KLF4 functions as a suppressor in HCC cell proliferation and migration. Consistent with its inhibition of cell proliferation, we also observed that ectopic KLF4 impaired aerobic glycolysis in HCC cells.

Given the important role of KLF4 in aerobic glycolysis in HCC cells, we further investigated the underlying mechanisms. KLF4 is reported to transcriptionally regulate the expression of LDHA in pancreatic cancer metabolism^30^. Based on the conception, we examined the effect of KLF4 on SIRT4 expression. Specifically, our results revealed that overexpression of KLF4 led to increased expression of SIRT4, and SIRT4 staining in HCC tissues was positively correlated with KLF4 staining, suggesting that KLF4 might be a transcriptional factor of SIRT4. The ChIP assay confirmed the binding of KLF4 to the SIRT4 promoter at its conserved critical region, suggesting that KLF4 transcriptionally activates SIRT4. As we previously showed that SET8 suppressed the transcription activity of SIRT4, we speculated that SET8 might restrain the effect of KLF4 on SIRT4. Notably, we found that SET8 suppressed the transcription activity of SIRT4 even in the presence of KLF4, whereas the mutant SET8 failed to affect the transcription activity. It appears that the interaction between SET8 and KLF4 is critical for SET8 to attenuate KLF4-mediated transcriptional activation of SIRT4, since mutations in SET8 abolished the inhibitory effect of SET8 itself. Previous studies demonstrated that DNA methylation is responsible for decreased KLF4 expression^52,53^. Whether SET8 regulates KLF4 expression through methylation or else needs our further efforts.

In summary, we reported the identification of SET8 as a significant regulator of the Warburg effect in hepatocellular carcinoma. SET8 directly or indirectly bound to and inactivated the expression of KLF4, and thus led to suppressed expression of its downstream SIRT4, resulting in a metabolic shift from oxidative phosphorylation to aerobic glycolysis. Together with other findings, we provided new evidences for the role of SET8 in glucose metabolism and delineated an unexpected SET8/KLF4/SIRT4 pathway regulating the Warburg effect required for HCC cell survival. This study highlights SET8 as a regulator of HCC progression and a worthy target for the development of new therapeutic strategies in the future.

## Supplementary information

Supplementary information

Supplementary Figure 1

Supplementary Figure 2

Supplementary Figure 3

Supplementary Figure 4

Supplementary Figure 5

TCGA cohort
