# Supplementary material for: Monomethyltransferase SET8 facilitates hepatocellular carcinoma growth by enhancing aerobic glycolysis
Source: Cell Death Dis. 2019 Apr 5;10(4):312. doi: 10.1038/s41419-019-1541-1 (PMC6450876; doi:10.1038/s41419-019-1541-1)
Supplement: Supplementary file 1 — Supplementary information [file 41419_2019_1541_MOESM1_ESM.docx]

**Supplementary Materials and Methods**

**Computational analysis of The Cancer Genome Atlas (TCGA) RNASeqV2 data**

Hepatocellular carcinoma RNASeqV2 data and clinical data were downloaded using TCGA Assembler. 370 Patients were classified into either low-gene expression group or high-gene expression group using median expression calculated by Cox regression analysis. Overall survival rates between high- and low-gene expression groups were assessed by Kaplan-Meier analysis and compared using the log-rank test. Statistical tests were two-sided and conducted with SPSS software.

**Oxygen consumption rate (OCR) and extracellular acidification rate (ECAR)**

Cellular mitochondrial function was determined with Seahorse XF Cell Mito Stress Kit and Bioscience XF96 Extracellular Flux Analyzer according to the manufacturer’s instructions. Cells were plated in XF96 Cell Culture Microplates at a cellular density of 20,000 cells/well. For OCR assay, cells were incubated with 175 μl Seahorse buffer (DMEM with phenol red containing 25 mmol/L glucose, 2 mmol/L sodium pyruvate, and 2 mmol/l glutamine) plus 25 μl each of 1 μmol/L oligomycin, 1 μmol/L FCCP, and 1μmol/L rotenone, prior to the measurement using the XF Cell Mito Stress Kit (Seahorse Bioscience, North Billerica, MA). For glycolytic assay, cells were incubated in a basal medium with 25 μl each of 10mmol/L glucose, 1 μmol/L oligomycin, and 100 mmol/L 2-deoxy-glucose prior to measurement using the Glycolytic Test Kit (Seahorse Bioscience, North Billerica, MA). Data were normalized to cell number and are plotted as mean ± SD.

**Co-immunoprecipitation (Co-IP) and immunoblotting**

Whole cell protein lysates for immunoprecipitation (IP) were extracted with cell lysis buffer for western and IP with PMSF (Beyotime Biotechnology, Shanghai). For endogenous IP, supernatants were incubated with corresponding primary antibodies and 50 μl protein A/G Dynabeads (ThermoFisher, USA) at 4℃ overnight. For exogenous IP, supernatants were incubated with anti-flag beads (Sigma, USA) at 4℃ overnight. Input and IP were then subjected to western blotting.

**Clinical Samples**

Tumor specimens and paired adjacent liver specimens were acquired during surgical resection on patients treated for HCC. The study contained 20 participants and each provided informed consent. All the procedures performed in this study were approved by the ethics committee of Fudan University.

**Immunohistochemistry (IHC)**

Standard IHC procedures were carried out with anti-KMT5A (Abcam, MA) and anti-KLF4 (Abcam, MA) antibodies. Tissues were embedded in paraffin, and processed for IHC. Sections were incubated with primary antibodies overnight at 4℃ in a humidified chamber. EnVision^TM^ Detction Kit (Glostrup, Denmark) was used to detect signals according to the manufacturer’s instructions with diaminobenzidine (DAB) as the enzyme substrate. IHC staining was scored by two investigators independently. The staining intensity was graded from 0 to 2 (0, no staining; 1, weak; 2, strong). Five different fields of each slide were randomly chosen for scoring. The proportion was graded from 0 to 4 (0% as 0, 1%-25% as 1, 26%-50% as 2, 51%-75% as3, and 76%-100% as 4). The staining intensity and staining proportion were combined to obtain a total positive score ranging from 0 to 8 (0-4 in a low level; 6-8 in a high level).

**Immunofluorescence (IF)**

Cells were seeded onto glass slides, washed with phosphate-buffered saline, fixed with 4% paraformaldehyde, permeabilized with 0.3% Triton X-100 for 5 min, and blocked for 1 h at room temperature. Cells were then stained with anti-KMT5A (Abcam, MA) and anti-KLF4 (Abcam, MA) antibodies overnight at 4℃. 4,6-diamidino-2-phenylindole (DAPI) was used to stain nucleus. Images were photographed with a confocal Leica fluorescence microscope.

**Protein extraction and western blot**

Whole cell protein lysates were harvested and protein concentrations were determined by a bicnchoninic acid protein assay kit (Beyotime Biotechnology, Shanghai). Primary antibodies against KMT5A (Abcam, MA), KLF4 (Abcam, MA), SIRT4 (Proteintech, Wuhan), GLUT1 (Proteintech, Wuhan), HK2 (Proteintech, Wuhan), LDHA (Proteintech, Wuhan), PGK1 (Proteintech, Wuhan), and a secondary antibody (anti-rabbit IgG or anti-mouse IgG, Proteintech, Wuhan) were used for western blotting. Equal amount protein sample loading was monitored and β-actin was used as a loading control.

**RNA extraction and quantitative real-time PCR (qRT-PCR)**

Total RNA was extracted with TRNzol regent according to the manufacturer’s instructions (TIANGEN BIOTECH CO., LTD, Beijing). Complementary DNA was synthesized with a TaKaRa PrimeScript regent kit. qRT-PCR with SYBR Green Master Mix was performed according to the manufacturer’s protocol. The primer sequences used were as follows: SET8 (F:5’-AGCTCCAGGAAGAGCAAAGCCGAG-3’; R:5’- GGCGTCGGTGATCTCGATGAGGT-3’); KLF4 (F: 5’-CTGCGGCAAAACCTACACAA-3’; R:5’-GCTCGCATTTTTGGCACTG-3’); SIRT4 (F:5’- ATGTGGATGCTTTGCACACCAAGG-3’; R:5’-TTCAGGACTTGGAAACGCTCTTGC-3’); GLUT1 (F:5’-CTTTGTGGCCTTCTTTGAAGT-3’; R:5’-CCACACAGTTGCTCCACAT-3’); HK2 (F:5’-GATTGTCCGTAACATTCTCATCGA-3’; R:5’-TGTCTTGAGCCGCTCTGAGAT-3’); LDHA (F:5’-TGGAGATTCCAGTGTGCCTGTATGG-3’; R:’;- CACCTCATAAGCACTCTCAACCACC-3’); PGK1 (F:5’-CAAGGTTAAAGCCGAGCCAGCCAA-3’; R:5’-GCCTTCTGTGGCAGATTGACTCC-3’); and β-actin (F: 5’-CTACGTCGCCCTGGACTTCGAGC-3’; R:5’-GATGGAGCCGCCGATCCACACGG-3’).

**Cell viability assay**

Cell viability was determined with Cell Counting Kit-8 (CCK-8; Dojindo Molecular Technologeis, Inc., Japan) according to the manufacturer’s instructions. Cells were grown onto a 96-well microplate with a density of 10^3^ cells/well. After corresponding treatments, cells were incubated with 10 μL of CCK-8 solution in 100 μL fresh medium for 2 h at 37℃. The optical density (OD) at 450 nm was measured and considered as an index of relative cell viability.

**Colony formation assay**

Cells were grown onto a 6-well plate with a density of 500 cells/well. After 7 to 14 days, cells were fixed with 4% paraformaldehyde for 15 min and stained with crystal violet (Sigma, USA) for 15 min at room temperature. A total number of more than 50 cells were considered as a valid colony. Colonies were photographed and counted from three independent experiments.

**siRNA transfection**

Human siRNAs were purchased from Proteintech. The sequences of siRNA against SIRT4 were: siRNA#1, sense, CCAGACUACAGGUCAGAAAdTdT; anti-sense, UUUCUGACCUGUAGUCUGGdTdT. siRNA#2, sense, CAGGUAUACUCUGGUUACAdTdT; anti-sense, UGUAACCAGAGUAUACCUGdTdT. Cells were transfected with siRNA using Lipofectamine 3000 (Invitrogen, USA) according to the manufacturer’s instructions.

**MicroPET/CT imaging**

MicroPET/CT imaging was performed and analyzed as described previously^1^.

**TUNEL assay**

TUNEL assay was performed using an In Situ Cell Death Detection Kit Fluorescence (Beyotime Biotechnology, Shanghai, China) according to the manufacturer’s instructions. The staining tissues were analyzed with a fluorescent microscope. The percentage of TUNEL positive cells were calculated using Image J in three random fields of each tissue from three independent staining.

**Supplementary Figure Legend**

Supplementary Fig. 1. SET8 contributed to tumor progression *in vitro*. (**a**) Overexpression of SET8 verified by real-time PCR and western blotting. (**b**) Knockdown of SET8 verified by real-time PCR and western blotting. (**c**) Assessment of cell growth via CCK-8 assay in control (Vector) and SET8 overexpression (SET8) M3 (left) and 97H (right) cells. (**d**) Migration assay of Vector and SET8 cells using a transwell system. Upper, M3 cells; down, 97H cells. (**e**) Colony formation assay performed in Vector and SET8 cells, and numbers of colonies were counted 14 days later. (**F**) Flow cytometry analysis using Annexin V and 7AAD staining to determine the apoptosis rate. Upper, M3 cells; down, 97H cells. Data are shown as mean ± SD of three independent experiments. ^*^*P*<0.05.

Supplementary Fig. 2. SET8 enhanced aerobic glycolysis in HCC cells. (**a** and **b**) Analysis of extracellular acidification rate (ECAR) and oxygen consumption rate (OCR) in M3 (left) and 97H (right) cells with either control vector or SET8 overexpression. (**c** and **d**) The expression levels of genes involved in glucose metabolism in indicated HCC cell lines with either control vector or SET8 overexpression detected by real-time PCR and western blot analysis. (**e**) SET8 shRNA infected cells were transfected with SET8^R259G^ plasmid for 48 hours, and cells were then subjected to western blot analysis. Data are shown as mean ± SD of three independent experiments. ^*^*P*<0.05.

Supplementary Fig. 3. SIRT4 silencing led to enhanced glycolysis in HCC cells. (**a**) Analysis of extracellular acidification rate (ECAR) in indicated HCC cell lines with SIRT4 silencing. Cells were transfected with NC or siRNAs against SIRT4, and 48 hours later cells were subjected to ECAR analysis. (**b** and **c**) The expression levels of SIRT4 and genes involved in glucose metabolism in indicated HCC cell lines with SIRT4 silencing detected by real-time PCR and western blot analysis. Cells were transfected with NC or siRNAs against SIRT4, and 48 hours later cells were harvested and subjected to real-time PCR and western blot analysis. (**d**) SET8-infected cells were transfected with SIRT4. Extracts were then subjected to western blot analysis. (**e-g**) SET8 knocking down cells were transfected with siRNA against SIRT4. Cells were subjected to ECAR, cell proliferation and apoptosis analysis. *compared with the control group; #compared with the shSET8 group. Data are shown as mean ± SD of three independent experiments. ^*^*P*<0.05. ^#^*P*<0.05.

Supplementary Fig. 4. KLF4 silencing led to enhanced glycolysis in HCC cells. (**a**) Protein level of KLF4 detected in SET8 overexpressing cells. (**b**) Validation of KLF4 overexpression by western blot analysis. (**c** and **d**) Analysis of extracellular acidification rate (ECAR) and oxygen consumption rate (OCR) in indicated HCC cell lines with KLF4 silencing. Cells were transfected with control shRNA or KLF4 shRNAs, and 48 hours later cells were subjected to ECAR analysis. (**e** and **f**) The expression levels of KLF4 and genes involved in glucose metabolism in indicated HCC cell lines with KLF4 silencing detected by real-time PCR and western blot analysis. Cells were transfected with control shRNA or KLF4 shRNAs, and 48 hours later cells were harvested and subjected to real-time PCR and western blot analysis. (**g-j**) SET8 knocking down cells were transfected with shKLF4 plasmid. Cells were then subjected to ECAR, OCR, cell proliferation and apoptosis analysis. *compared with the control group; #compared with the shSET8 group. Data are shown as mean ± SD of three independent experiments. ^*^*P*<0.05. ^#^*P*<0.05.

Supplementary Fig. 5. KLF4 silencing supported the growth of HCC cells. (**a**) Assessment of cell growth via CCK-8 assay in indicated HCC cell lines with either control shRNA or KLF4 shRNAs. (**b**) Migration assay of control and KLF4 knockdown cells using a transwell system. Upper, M3 cells; down, 97H cells. (**c**) Colony formation assay performed in indicated HCC cell lines with either control shRNA or KLF4 shRNAs, and numbers of colonies were counted 14 days later. (**d**) Flow cytometry analysis using Annexin V and 7AAD staining to determine the apoptosis rate. Upper, M3 cells; down, 97H cells. (**e**) Assessment of cell growth via CCK-8 assay in indicated HCC cell lines with either KLF4 overexpression or control vector. (**f**) Migration assay of KLF4 overexpression and control cells using a transwell system. Upper, M3 cells; down, 97H cells. (**g**) Colony formation assay performed in indicated HCC cell lines with either KLF4 overexpression or control vector, and numbers of colonies were counted 14 days later. (**h**) Flow cytometry analysis using Annexin V and 7AAD staining to determine the apoptosis rate. Upper, M3 cells; down, 97H cells. Data are shown as mean ± SD of three independent experiments. ^*^*P*<0.05.

**Supplementary References**

1. Chen X, Wu Q, Sun P, Zhao Y, Zhu M, Miao C. Propofol Disrupts Aerobic Glycolysis in Colorectal Cancer Cells via Inactivation of the NMDAR-CAMKII-ERK Pathway. *Cell Physiol Biochem* 2018, **46**(2)**:** 492-504.
