# Supplementary figures and images for: Monomethyltransferase SET8 facilitates hepatocellular carcinoma growth by enhancing aerobic glycolysis
Source: Cell Death Dis. 2019 Apr 5;10(4):312. doi: 10.1038/s41419-019-1541-1 (PMC6450876; doi:10.1038/s41419-019-1541-1)

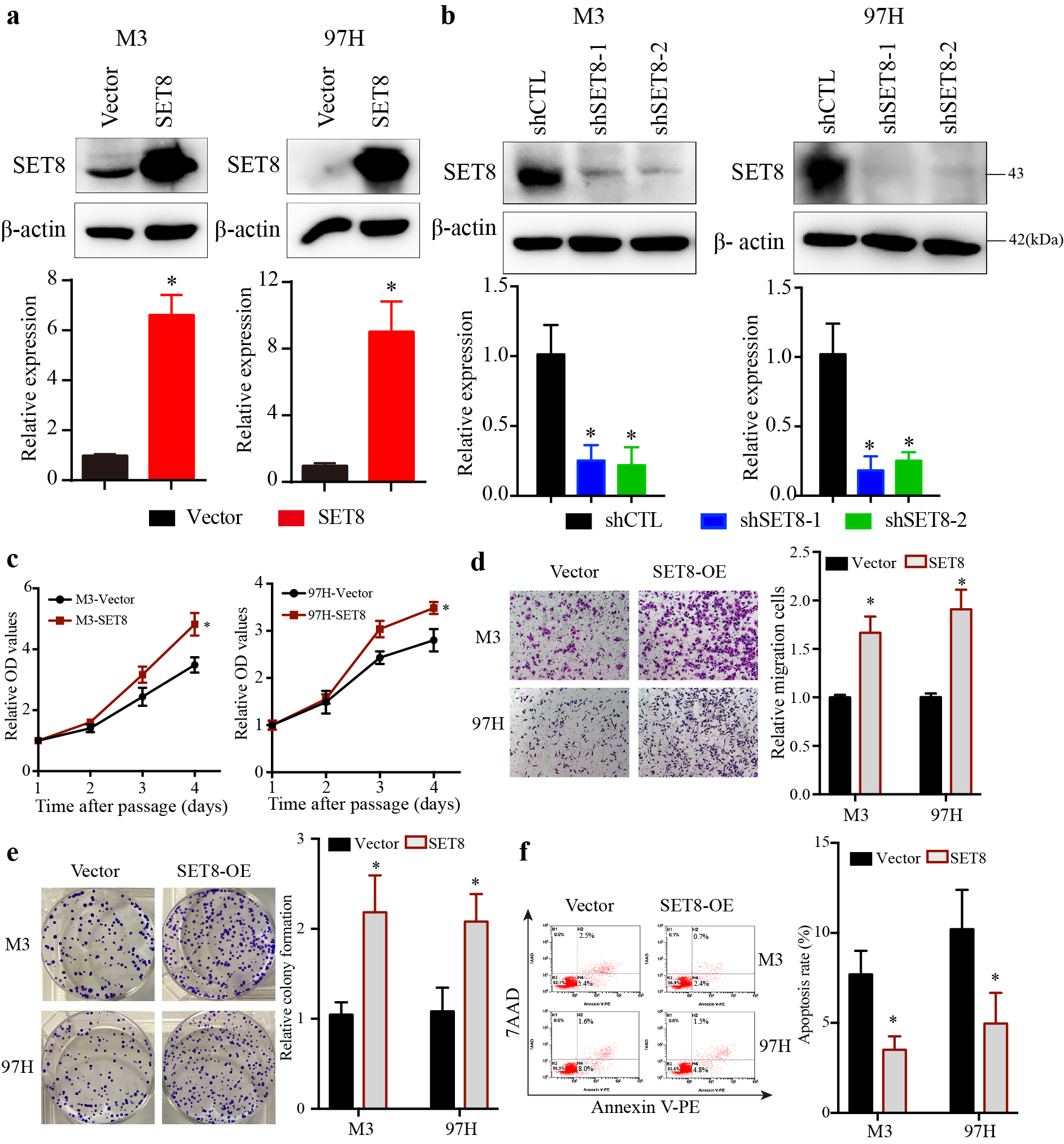

Supplement: Supplementary file 2 — Supplementary Figure 1 [file 41419_2019_1541_MOESM2_ESM.tif]

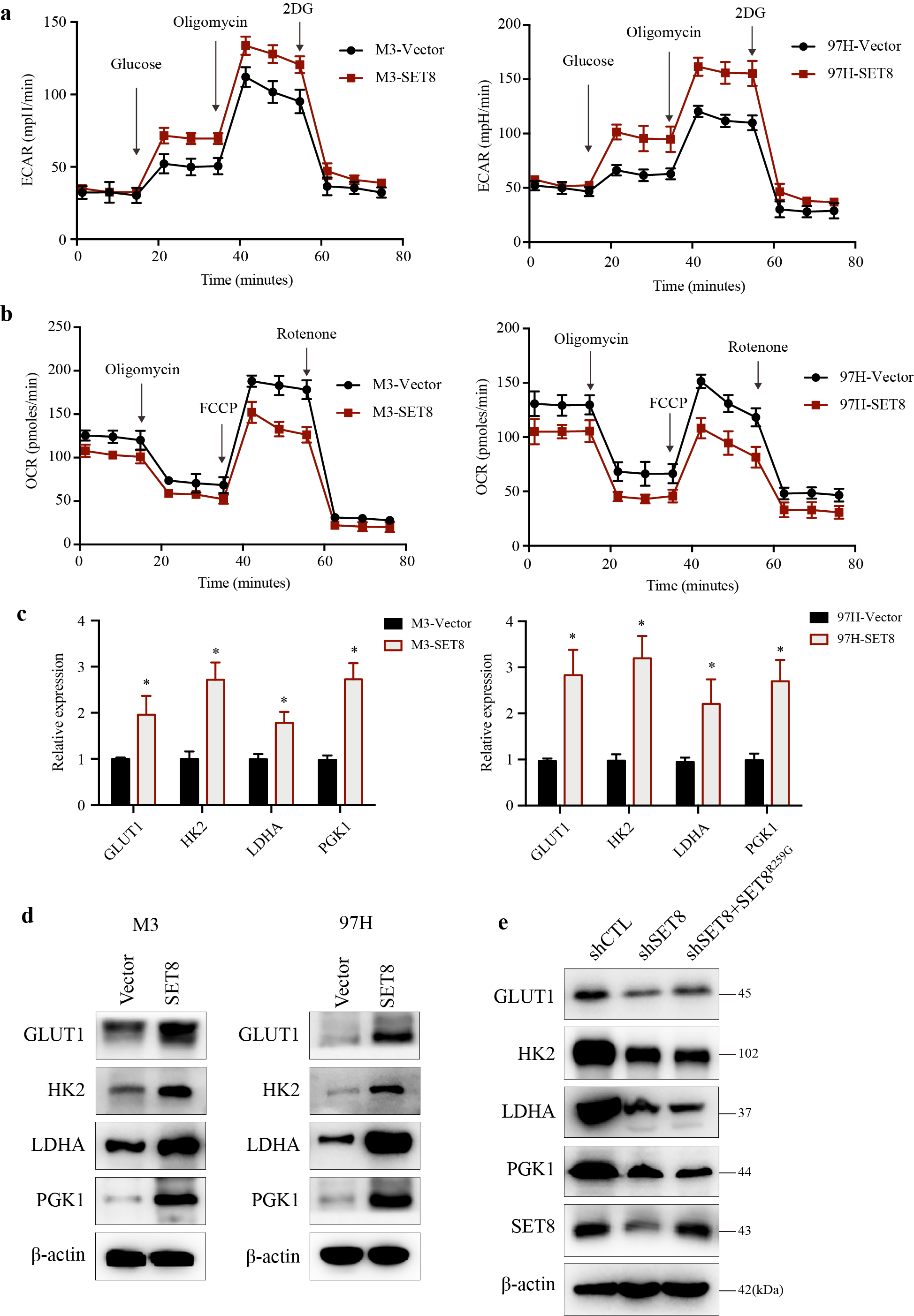

Supplement: Supplementary file 3 — Supplementary Figure 2 [file 41419_2019_1541_MOESM3_ESM.tif]

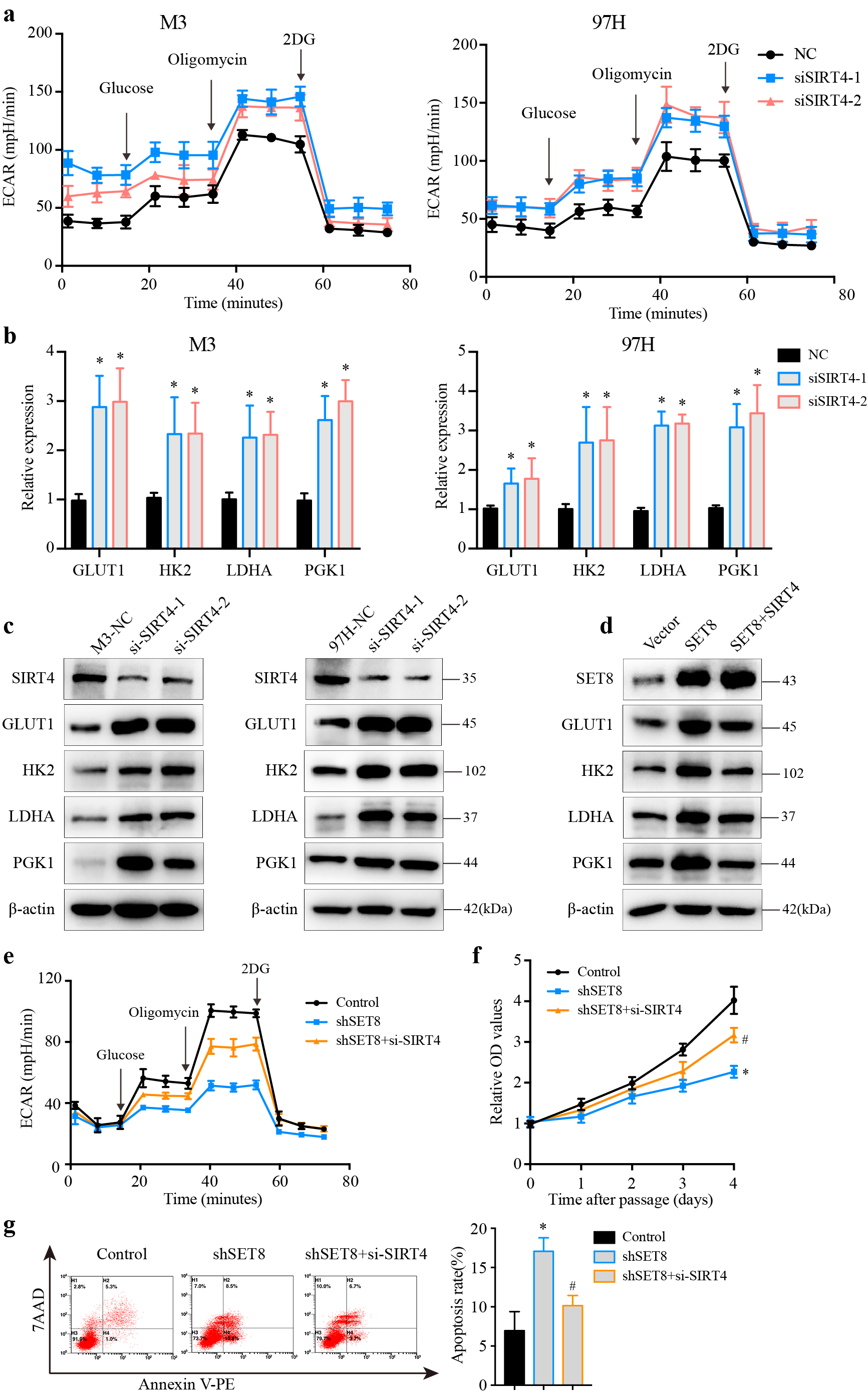

Supplement: Supplementary file 4 — Supplementary Figure 3 [file 41419_2019_1541_MOESM4_ESM.tif]

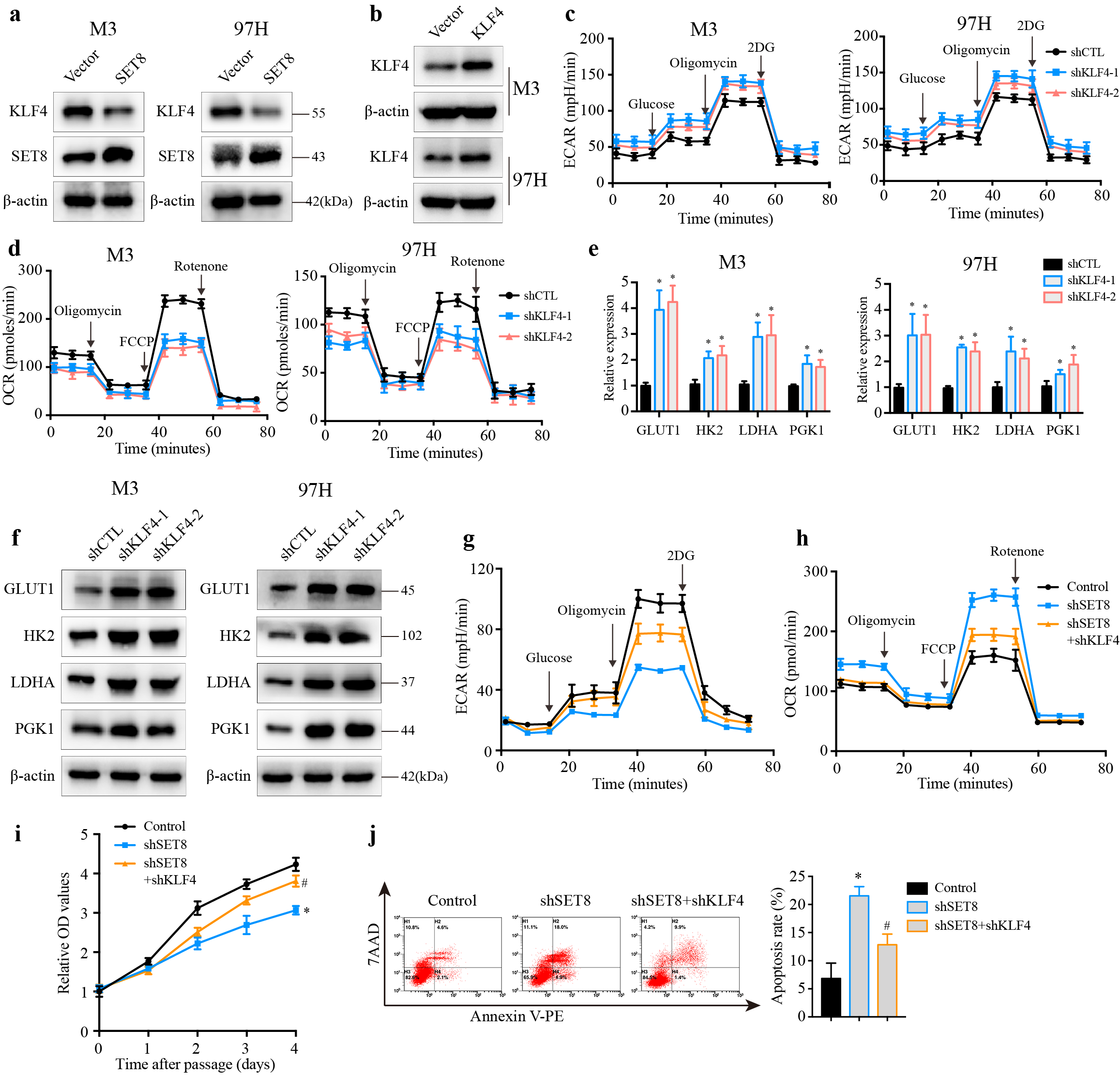

Supplement: Supplementary file 5 — Supplementary Figure 4 [file 41419_2019_1541_MOESM5_ESM.tif]

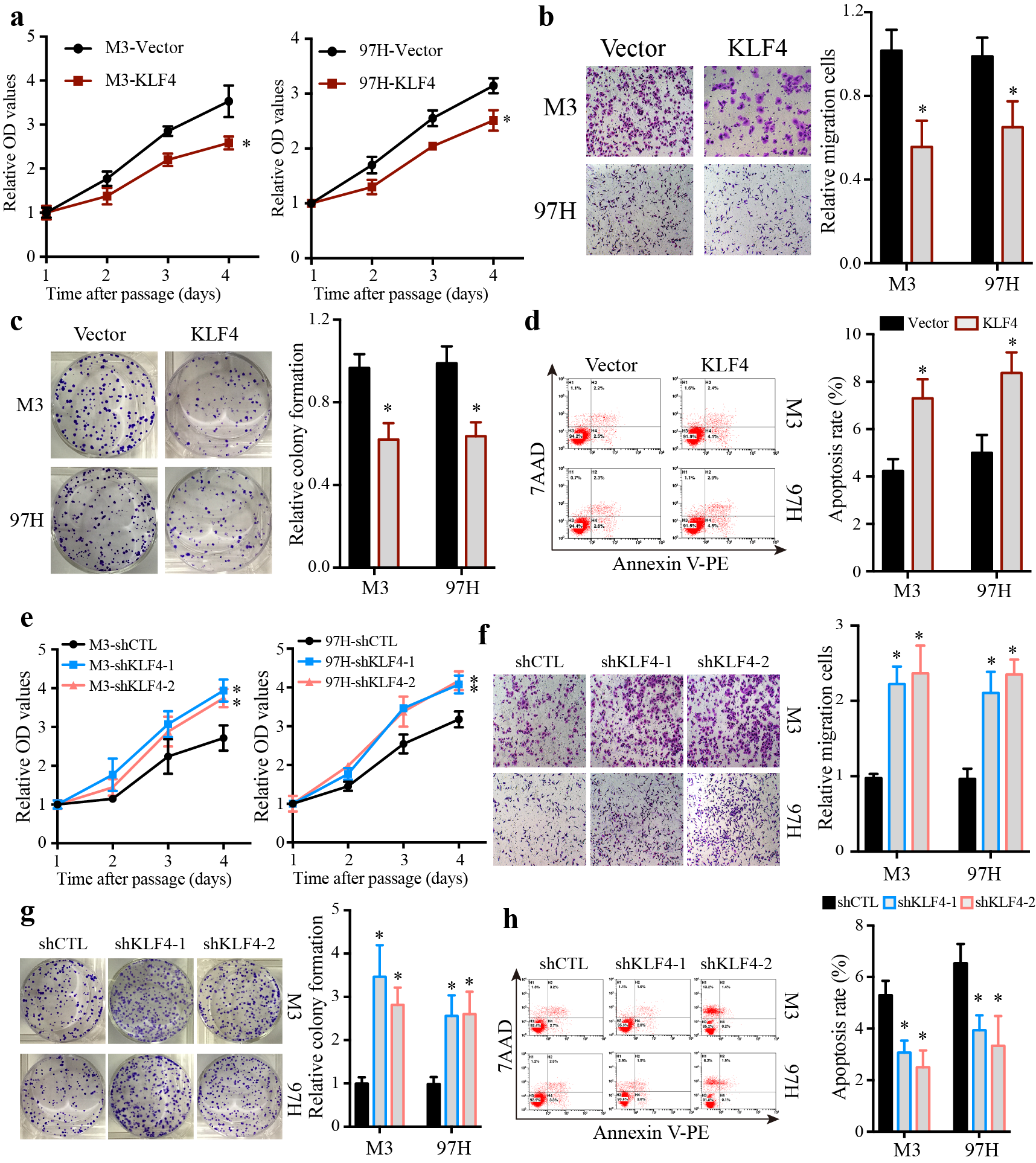

Supplement: Supplementary file 6 — Supplementary Figure 5 [file 41419_2019_1541_MOESM6_ESM.tif]
